# Photoinduced Redox Initiation System for Delayed-Onset Radical UV-Curable Adhesives on Opaque Substrates

**DOI:** 10.1021/acsmacrolett.6c00416

**Published:** 2026-09-28

**Authors:** Naoya Machida, Ataru Daimon, Daisuke Aoki, Koji Arimitsu

**Affiliations:** Department of Pure and Applied Chemistry, 13258 Tokyo University of Science, 2641 Yamazaki, Noda, Chiba 278-8510, Japan

## Abstract

Conventional radical ultraviolet (UV) curing requires optically transparent substrates owing to the rapid termination of propagating radicals, limiting its use in opaque or geometrically complex assembly cases. We report a photoinduced redox initiation system for delayed-onset radical UV-curable adhesives combining a coumarin-based photobase generator (Cou-TMG) with dilauroyl peroxide (LP). Upon UV irradiation, Cou-TMG releases 1,1,3,3-tetramethylguanidine, which reacts with LP through a redox process that continuously generates radicals, sustaining polymerization after irradiation stops. Optimized photobase generator loading and irradiation dose provide an open time sufficient for substrate lamination (low C=C conversion immediately after UV irradiation) and endow substantial postpolymerization capability (higher C=C conversion after 1 h), contrasting conventional radical UV curing systems. The delayed-onset, radical UV-curable adhesives are applicable to various multifunctional acrylates. Specifically, trimethylolpropane triacrylate-based adhesives achieve a high lap-shear strength of 4.4 MPa on aluminum, and the present system further bonds Cu, Fe, polycarbonate, and glass substrates.

## Graphical abstract

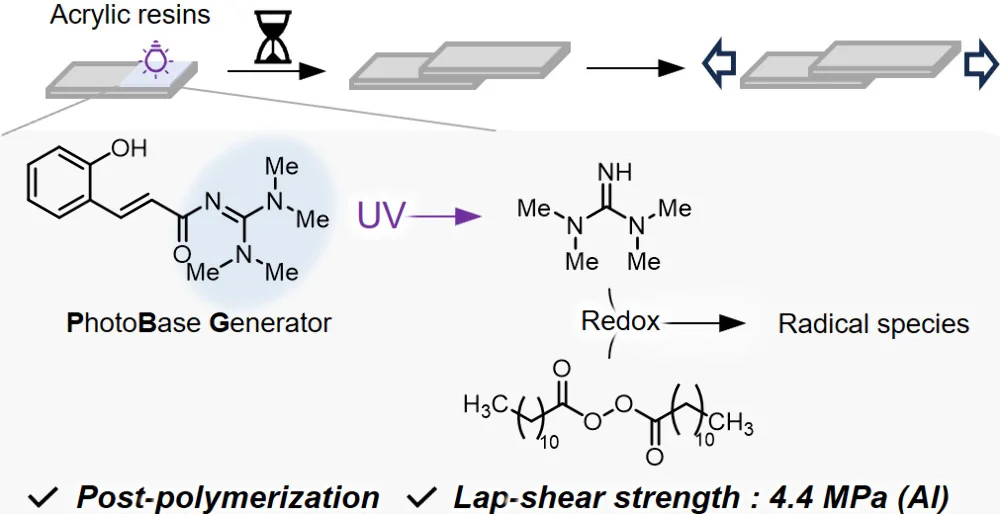

Radical ultraviolet (UV) curing of acrylic resins has been extensively used in diverse fields, including healthcare, automotive, and electronics, owing to its rapid polymerization kinetics.^1–6^ Radical UV-curable adhesives operate at room temperature, making them well-suited for heat-sensitive substrates.^3^ However, a critical limitation of this technology is that direct light penetration is required to initiate polymerization.^1,7^ Consequently, its use is largely restricted to optically transparent materials; opaque substrates, such as metals, block light transmission and thereby impede bonding. As a result, adhesion to opaque substrates still relies on thermally cured adhesives or pressure-sensitive adhesives based on physical cross-linking.^8,9^

Delayed-onset UV-curable adhesives have emerged as a promising strategy for bonding opaque substrates.^10^ In this approach, substrates are brought into contact after UV irradiation. The resin must therefore exhibit “post-polymerization” capability—the ability to continue reacting after the light is switched off—to provide sufficient “open time” for assembly.^11^ Cationic and anionic systems (the latter often based on photobase generators, PBGs) are known to support this type of delayed-onset curing because their ionic active species are relatively long-lived,^10,12–16^ whereas radical systems typically lack this capability. In conventional radical polymerization, radical species have short lifetimes and terminate rapidly once irradiation stops.^17^ Although some radical systems show limited postpolymerization capability under specific viscosity or irradiation conditions, these effects are typically insufficient for practical delayed-onset adhesives.^11,18^ Consequently, the development of robust radical-based, delayed-onset UV-curable adhesives for opaque substrates remains a major scientific challenge.

To address this challenge, a photoinduced redox initiation system is desirable. Redox initiators, which typically comprise a reducing agent, such as an amine or guanidine, and an oxidizing agent, such as an organic peroxide, generate radicals at room temperature.^19–21^ Kim et al. reported that radical generation in amine–peroxide systems is a relatively slow process governed by the rate-limiting homolysis of intermediates.^19^ Photoresponsiveness has been imparted to these types of systems by using PBGs to mask the basic component.^22–25^ Notably, He et al. demonstrated photoinduced redox initiation using a PBG and an organic peroxide.^24^ In their system, however, photolysis of the PBG itself generates radical species in addition to releasing the base, so that direct photoradical initiation contributes to polymerization during irradiation. Although this is advantageous for rapid photopolymerization, it is less suited to delayed-onset adhesion, for which polymerization during irradiation must be minimized to preserve sufficient fluidity for subsequent substrate assembly. Delayed-onset adhesion therefore benefits from a PBG-based redox system in which photobase generation is separated from radical generation. To the best of our knowledge, such a system has not previously been applied to delayed-onset UV-curable adhesives.

Herein, we demonstrate a delayed-onset radical UV-curable system enabled by a photoinduced redox initiator. We employed a coumarin-based photobase generator (Cou–TMG) that releases 1,1,3,3-tetramethylguanidine (TMG), a strong organic base, upon irradiation at 365 nm.^26^ A key advantage of Cou-type PBGs is that they do not release carbon dioxide upon activation, which is essential to avoid adhesion failure caused by gas evolution. The photogenerated TMG subsequently undergoes a redox reaction with dilauroyl peroxide (LP) to produce radicals continuously, even after irradiation is stopped (Figure 1). This mechanism affords sustained radical polymerization and thereby enables delayed-onset adhesion. In this study, we optimized the base structure of the redox system and evaluated the delayed-curing performance across a range of acrylic resins by using lap-shear strength tests. These findings provide a framework for extending radical UV bonding to opaque substrates.

**Figure 1:**
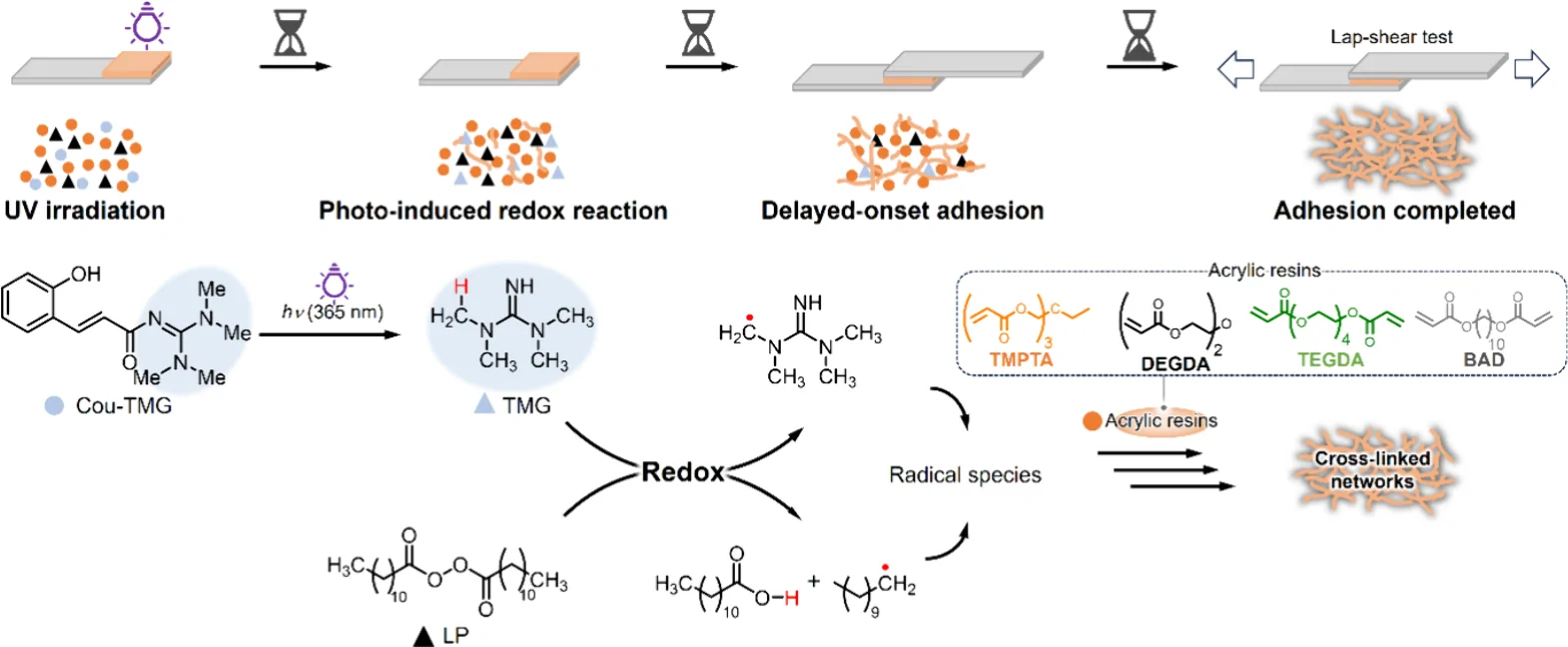
Schematic of the photoinduced redox initiation mechanism for delayed-onset ultraviolet (UV)-curable adhesion.

To identify the most effective base for the redox initiation system, we compared the delayed-onset radical polymerization behavior of a series of organic bases on CaF_2_ substrates using Fourier transform infrared spectroscopy (FT-IR) analyses (Figures 2a and S1). The candidate bases—TMG, *N,N*′-dicyclohexyl-4-morpholinecarboxamidine (DCMC), 1,5,7-triazabicyclo[4.4.0]­dec-5-ene (TBD), piperidine (Pip), and morpholine (Mor)—were selected because each possesses an N–H bond that can be functionalized to form a Cou-type PBG.^27^ The reaction mixtures consisted of tetrahydrofurfuryl acrylate (THFA), LP, and the organic base in a 100:1:1 molar ratio; THFA served as the monomer and solvent because it dissolves the redox components well. FT-IR spectra were recorded immediately after sample preparation to track the consumption of the acrylate C=C bonds. Only the TMG system exhibited a considerable increase in conversion over the 60 min monitoring period, reaching 80%. By contrast, the DCMC, TBD, Pip, and Mor systems exhibited almost no polymerization.

**Figure 2:**
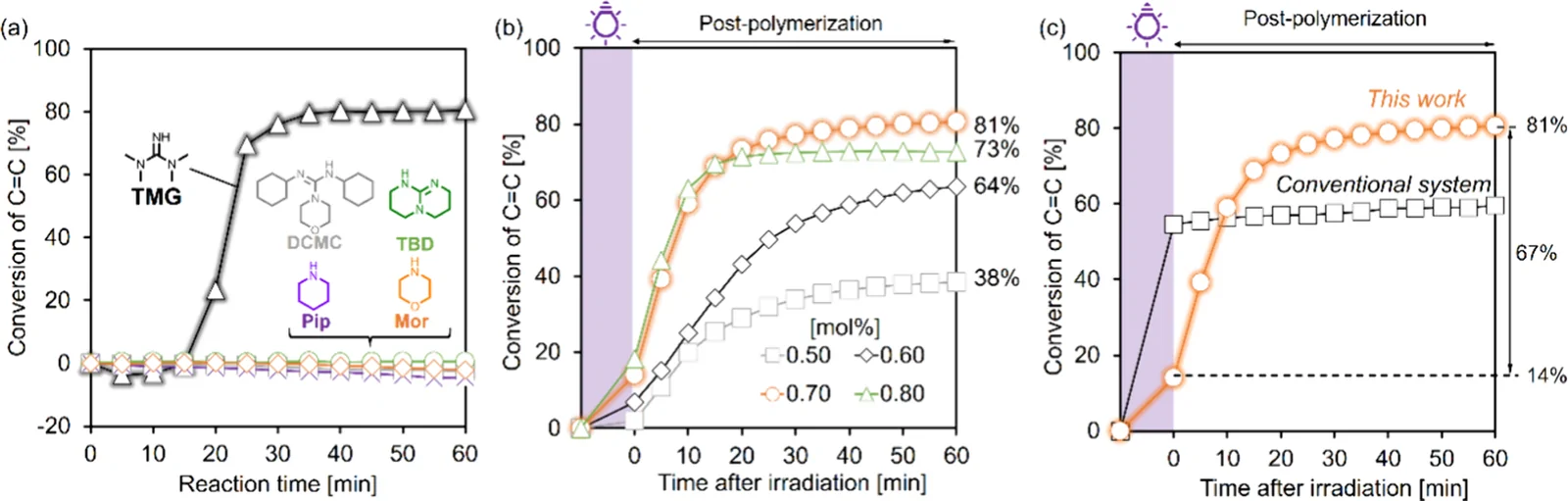
Polymerization behavior of the photoinduced redox initiation system. (a) Time-dependent C=C conversion of tetrahydrofurfuryl acrylate (THFA) in the presence of different organic bases (1,1,3,3-tetramethylguanidine (TMG), *N*,*N*′-dicyclohexyl-4-morpholinecarboxamidine (DCMC), 1,5,7-triazabicyclo[4.4.0]­dec-5-ene (TBD), piperidine (Pip), or morpholine (Mor)) with dilauroyl peroxide (LP), monitored by Fourier transform infrared spectroscopy (FT-IR) on CaF_2_ substrates. (b) Conversion profiles at various coumarin-based photobase generator (Cou-TMG) loadings in THFA/TMPTA. (c) Comparison of postirradiation conversion profiles between the photoinduced redox system (Cou-TMG/LP) and a conventional photoradical system (Omnirad 819).

These differences reflect the molecular structures of the organic bases. Kim et al. reported that in redox reactions with peroxides, secondary amines preferentially form stable byproducts that inhibit radical generation, whereas tertiary amines generate radicals efficiently.^19^ Radical generation was therefore suppressed in the Pip and Mor systems, both of which possess only secondary amine functionality. Among the amidine/guanidine-type bases (TMG, DCMC, TBD), TMG exhibited markedly higher reactivity than DCMC or TBD. Redox initiation is generally triggered by nucleophilic attack of the base on the peroxide. TBD, however, was much less reactive than TMG despite comparable nucleophilicity. Nucleophilicity alone thus cannot explain the reactivity, pointing to other factors such as competing reactions between the base and LP. A (2,2,6,6-tetramethylpiperidin-1-yl)­oxyl trapping experiment confirmed continuous radical generation in the TMG/LP system (Figure S2). To further exclude the possibility of base-catalyzed anionic polymerization, a control experiment was performed using the same formulation without LP. No appreciable increase in C=C conversion was observed after irradiation (Figure S9), indicating that the postpolymerization in the Cou-TMG/LP system originates predominantly from the redox radical mechanism rather than base-catalyzed anionic polymerization. On the basis of these findings, TMG was selected as the base for the redox initiator and was converted into the corresponding Cou-type PBG following a previously reported procedure.^10^

Subsequently, we evaluated the delayed-onset UV-curing behavior of Cou–TMG. A mixture containing Cou-TMG (0.50–0.80mol % relative to C=C), LP (0.80 mol % relative to C=C), and THFA/trimethylolpropane triacrylate (TMPTA) (1:1 C=C ratio) was prepared. In this series, the LP loading was fixed while the Cou-TMG loading was varied; therefore, the Cou-TMG/LP ratio was not constant. The samples were irradiated with light at 365 nm (100 mW/cm^2^, 1.0 J/cm^2^), and the reaction progress was monitored on CaF_2_ substrates using FT-IR (Figures 2b, S3, and S4). Immediately after exposure, the C=C conversion remained below 20% under all conditions, ensuring that the resin retained sufficient fluidity for the bonding step. After 60 min, the final C=C conversions at Cou-TMG loadings of 0.50, 0.60, 0.70, and 0.80 mol % were 38%, 64%, 81%, and 73%, respectively, with a maximum at 0.70 mol %. The slightly lower final conversion at 0.80 mol % may arise from an inner-filter effect at the increased Cou-TMG concentration, which reduces the effective penetration of incident light. Because a low initial conversion is desirable to maintain the resin fluid and tacky during substrate lamination, 0.70 mol % was chosen as the optimal Cou-TMG loading.

To assess whether the low initial conversion observed by FT-IR translates into a practical working time for substrate lamination, the physical state and workability of the resin were examined visually after UV irradiation (Figures S6–S8 and Table S1). The resin remained highly fluid and could be readily laminated for up to 10 min after irradiation, whereas the viscosity increased noticeably at longer standing times. Under the present conditions, the resin therefore remained practically workable for substrate lamination for at least 10 min. These results demonstrate the feasibility of delayed-onset UV adhesion with this system.

To highlight the benefit of the present system for postpolymerization, we compared its behavior with that of a conventional system based on a photoradical initiator (PI), bis­(2,4,6-trimethylbenzoyl)­phenylphosphine oxide (Omnirad 819) (Figures 2c and S9). A mixture of Omnirad 819 (1.0 mol % relative to C=C) and THFA/TMPTA (1:1 C=C ratio) was prepared, and the reaction progress after UV irradiation was monitored using FT-IR. The PI-based system showed only an approximate 5% increase in conversion during the 60 min after irradiation, whereas the redox system showed a 67% increase (from ∼14% to ∼81%). This substantial postpolymerization confirms the utility of the proposed photoinduced redox initiation system.

To determine the optimal irradiation dose for delayed-onset UV adhesion, we examined the polymerization behavior as a function of exposure energy. Using the optimized formulation, samples were irradiated with light at 365 nm (100 mW/cm^2^) at the doses of 0, 0.10, 0.50, and 1.0 J/cm^2^, and the reaction progress was monitored using FT-IR (Figures 3a and S10). No polymerization occurred at 0 or 0.10 J/cm^2^, whereas substantial polymerization proceeded at 0.50 and 1.0 J/cm^2^, reaching C=C conversions of 65% and 81%, respectively, after 60 min. Because the conversion nearly leveled off at 1.0 J/cm^2^, this dose was selected as optimal for providing sufficient radical generation.

**Figure 3:**
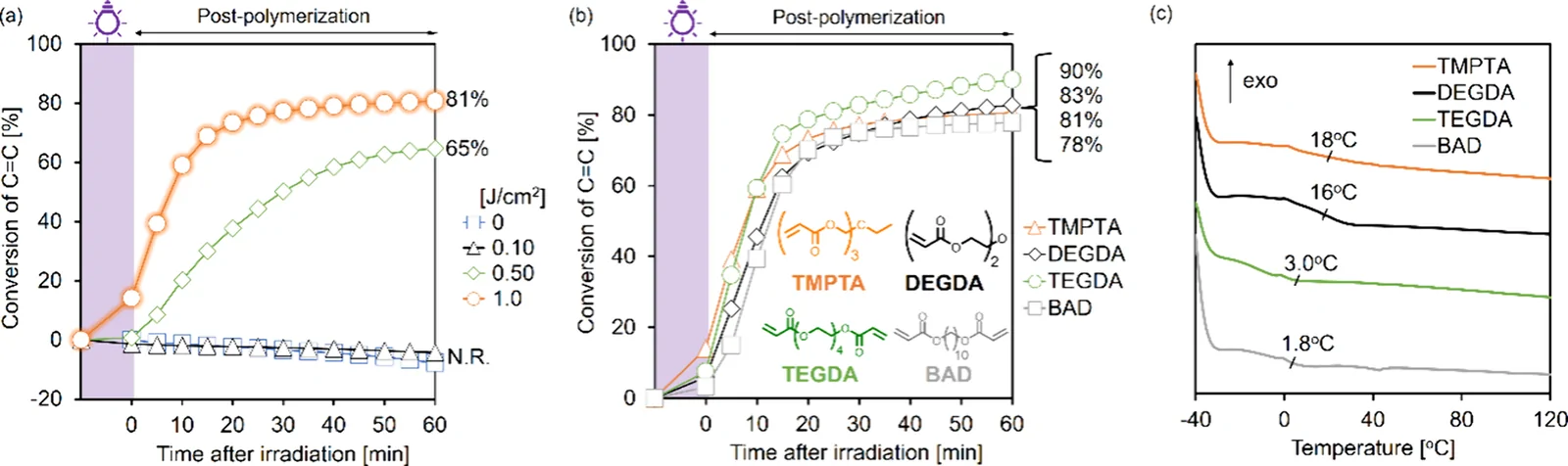
Photoinduced redox polymerization behavior. (a) Time-dependent C=C conversion of THFA/TMPTA after UV irradiation at different doses (0, 0.10, 0.50, and 1.0 J/cm^2^), monitored by FT-IR. (b) Time-dependent C=C conversion of THFA combined with different multifunctional acrylates (TMPTA, DEGDA, TEGDA, and BAD) after UV irradiation. (c) Differential scanning calorimetry thermograms of the cured THFA combined with different multifunctional acrylates (TMPTA, DEGDA, TEGDA, and BAD).

The versatility of the photoinduced redox initiation system was evaluated with several multifunctional acrylates: TMPTA, diethylene glycol diacrylate (DEGDA), tetra­(ethylene glycol) diacrylate (TEGDA), and 1,10-bis­(acryloyloxy)­decane (BAD) (Figures 3b and S11). All tested monomers underwent polymerization, confirming the broad applicability of the present strategy. They reached similar final C=C conversions after 60 min (TEGDA, 90%; DEGDA, 83%; TMPTA, 81%; BAD, 78%), with the small variation likely reflecting differences in backbone polarity. A similar polarity effect has been reported for amine–peroxide redox systems, in which the polarity of the reaction environment affects the stability of intermediates and the overall reaction rate.^20^ A more polar environment may stabilize these intermediates, thereby accelerating radical generation.

The thermal properties of the cured resins were characterized by their glass transition temperatures (*T*_g_) (Figure 3c). The *T*_g_ values were 18 and 16 °C for the TMPTA and DEGDA systems, respectively, whereas the TEGDA and BAD systems exhibited much lower values equal to 3.0 and 1.8 °C, respectively. These differences reflect the monomer’s structure whereby longer molecular chains yield more flexible networks and therefore lower *T*_g_.

To further evaluate the practical applicability of the present system, we investigated the storage stability of the one-component adhesive formulation. The optimized TMPTA-containing formulation was stored at 25 °C under light-shielded conditions, and its appearance and fluidity were monitored over time. For comparison, an otherwise identical formulation containing TMG instead of Cou-TMG was also examined. Table 1 summarizes the appearance of the two formulations after storage. The Cou-TMG formulation remained liquid even after 7 days, whereas the TMG formulation, although liquid immediately after mixing, cured within 20 min. These results demonstrate that masking the base as a PBG improves the storage stability of the adhesive formulation. Delayed-onset UV adhesion was evaluated on aluminum (Al) substrates pretreated with 3-(trimethoxysilyl)­propyl acrylate (TSA) to enhance interfacial bonding (Figures 4a, S12, and S13). All formulations achieved successful adhesion, and all specimens showed interfacial failure at the Al/adhesive boundary (Table S2). Although radical-based systems typically suffer from rapid curing that is incompatible with delayed-onset adhesion, the present system overcomes this limitation through the distinctive kinetics of its redox initiation. The slow intrinsic radical generation and the continued radical supply after irradiation together provide a sufficient open time for assembly. Once the substrates are brought into contact, the exclusion of atmospheric oxygen further promotes polymerization within the bond line. The lap-shear strengths of the TMPTA and DEGDA systems were 4.4 and 3.3 MPa, respectively, whereas those of the TEGDA and BAD systems were both ∼1.3 MPa. This trend correlates with the *T*_g_ of the cured resins, indicating that higher network rigidity (higher *T*_g_) leads to better mechanical performance of the adhesive joint.

**Table 1:**
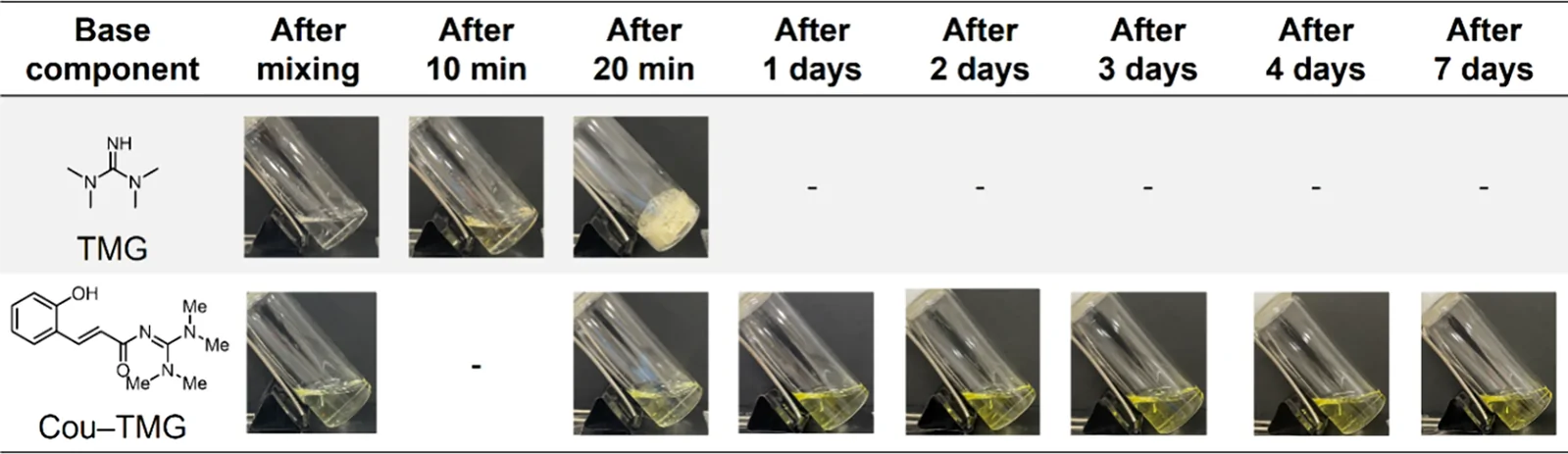
Appearance of the Cou-TMG and TMG One-Component Adhesive Formulations During Storage at 25 °C under Light-Shielded Conditions^a^

a Adhesive formulation: THFA/TMPTA (1:1 C=C ratio), LP (0.80 mol % relative to C=C), and TMG or Cou–TMG (0.70 mol % relative to C=C).

**Figure 4:**
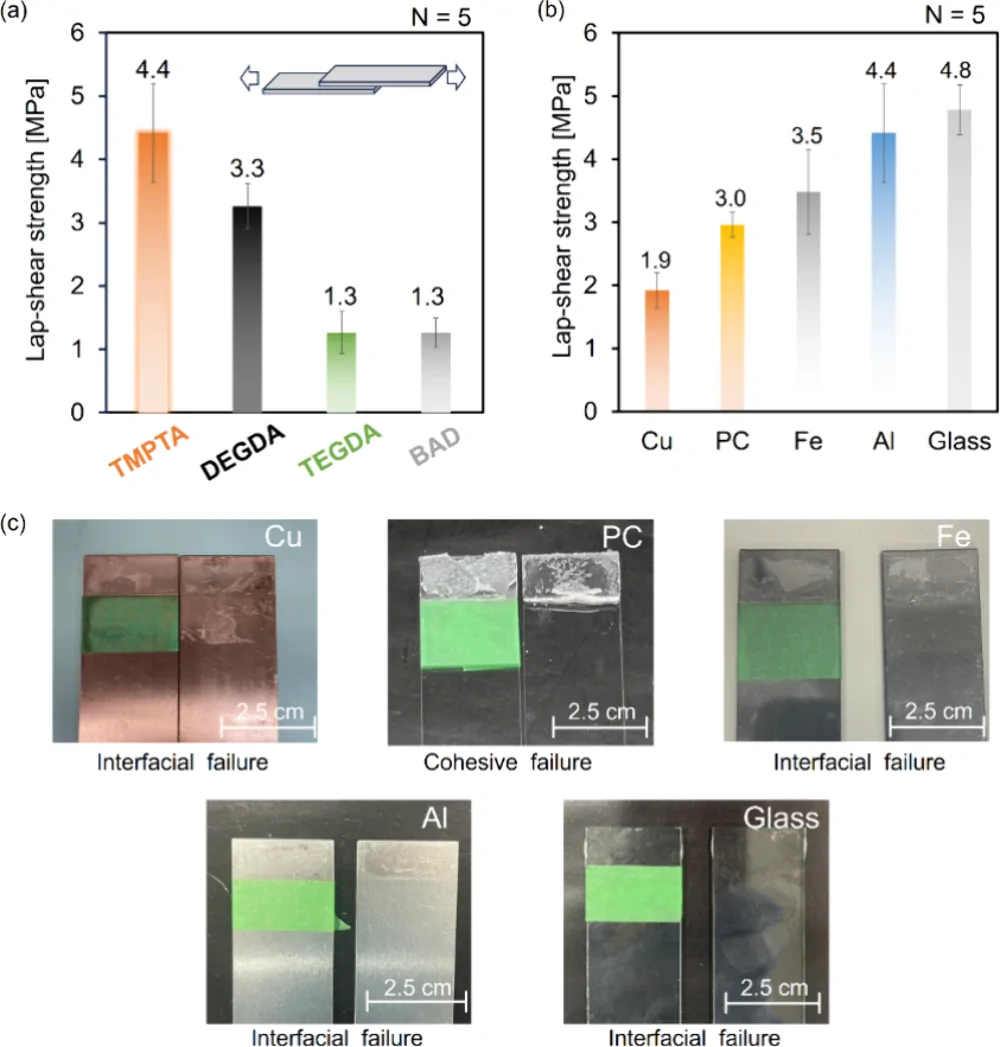
Delayed-onset UV adhesion performance of the THFA/TMPTA-based photoinduced redox initiation system on various substrates. (a) Lap-shear strength of aluminum substrates bonded with THFA combined with different multifunctional acrylic resins (*N* = 5). (b) Lap-shear strengths of Cu, PC, Fe, Al, and glass substrates bonded with the THFA/TMPTA adhesives based on delayed-onset UV adhesion (*N* = 5). (c) Photographs of the bonded specimens after lap-shear testing.

To assess the versatility of the present system, delayed-onset UV bonding was performed on various substrates (Cu, polycarbonate (PC), Fe, and glass) using the TMPTA-based formulation, which yielded the highest adhesive strength on Al (Figure 4b,c). The Cu, Fe, and glass substrates were pretreated with TSA in the same way as the Al substrates. The adhesive strengths and corresponding failure modes were 1.9 MPa (interfacial failure) for Cu, 3.0 MPa (cohesive failure) for PC, 3.5 MPa (interfacial failure) for Fe, and 4.8 MPa (interfacial failure) for glass (Table S3). Water contact angle measurements confirmed that TSA treatment altered the surface wettability of the metal substrates (Figure S14). Among the substrates examined, Al gave the highest adhesive strength. Although the full order of adhesive strength (Al > Fe > Cu) cannot be explained by the contact angle measurements alone, the particularly high value for Al may be related to differences in the chemical nature of the surface oxide layers and in the density and distribution of surface hydroxyl groups. Al is known to form a stable oxide layer with a high density of surface hydroxyl groups, which can favor the interaction and immobilization of silane coupling agents at the interface.^28^ Fe and Cu, in contrast, have been reported to form more heterogeneous oxide states with less uniform hydroxyl distributions, which may contribute to their lower adhesive strengths.^29^ These results demonstrate that the present photoinduced redox system enables effective delayed-onset UV adhesion on a broad range of substrates, including opaque metals that conventional radical UV adhesives cannot bond.

In summary, we have developed a delayed-onset radical UV-curable adhesion system enabled by a photoinduced redox initiator composed of a coumarin-type photobase generator (Cou-TMG) and LP. Photogenerated TMG reacts with LP to continuously produce radicals after irradiation, achieving robust postpolymerization. The system is applicable to a range of multifunctional acrylates (TMPTA, DEGDA, TEGDA, and BAD), with the *T*_g_ of the cured resin governing the adhesive strength. On aluminum, the TMPTA-based formulation reached a lap-shear strength of 4.4 MPa, and successful bonding was also achieved on Cu, Fe, PC, and glass. The trend across metals (Cu < Fe < Al) reflects differences in surface hydroxyl density and silane coupling efficiency, while the PC and glass substrates yielded cohesive and interfacial failures, respectively, confirming that the adhesive layer itself is sufficiently strong. By eliminating the need for continuous irradiation and optically transparent substrates, this strategy extends radical UV adhesion to opaque and geometrically complex assemblies.

## Supplementary Material



## Notes

FT-IR spectra; Lap-shear test; Water contact angles (PDF)
