# Supplementary material for: Photoinduced Redox Initiation System for Delayed-Onset Radical UV-Curable Adhesives on Opaque Substrates
Source: ACS Macro Lett. 2026 Sep 28;15(10):1532–7. doi: 10.1021/acsmacrolett.6c00416 (PMC13689913; doi:10.1021/acsmacrolett.6c00416)
Supplement: Supplementary file 1 [file mz6c00416_si_001.pdf]

Supporting Information

Photo-induced Redox Initiation System for Delayed-onset Radical UV-curable Adhesives on Opaque Substrates

*Naoya Machida, Ataru Daimon, Daisuke Aoki, Koji Arimitsu**

Department of Pure and Applied Chemistry, Tokyo University of Science, 2641 Yamazaki, Noda, Chiba 278-8510, Japan

*Email: arimitsu@rs.tus.ac.jp

Table of contents

1 Materials and Instruments S3

2 FT-IR and UV-vis measurements S4

3 Surface treatment of Al substrates..S16

4 Lap-shear Performance of Various Multifunctional Acrylates S17

5 Surface Treatment of Other Substrates (Cu, Fe, Al, and Glass).......................S18

6 Adhesion Performance of the THFA/TMPTA System on Various Substrates..S19

7 Reference..S19

1 Materials and Instruments

1.1 Materials

1,5,7-Triazabicyclo[4.4.0]dec-5-ene, piperidine, morpholine, tetrahydrofurfuryl acrylate (THFA), trimethylolpropane triacrylate (TMPTA), diethylene glycol diacrylate, 1,10-bis(acryloyloxy)decane, 3-(trimethoxysilyl)propyl acrylate (TSA) and 2,2,6,6-tetramethylpiperidine 1-oxyl (TEMPO) were purchased from Tokyo Chemical Industry Co., Ltd. 1,1,3,3-Tetramethylguanidine (TMG), tetra(ethylene glycol) diacrylate (TEGDA), and bis(2,4,6-trimethylbenzoyl)phenyl phosphine oxide (Omnirad 819) were purchased from Sigma–Aldrich. Dilauroyl peroxide (LP) was purchased from Nacalai Tesque, Inc. Methanol (super dehydrated) and tetrahydrofuran were purchased from Wako Pure Chemical Industries, Ltd. coumarin-based TMG (Cou–TMG) was synthesized following our previously reported procedure.[1]

1.2 Instruments

^{1}H nuclear magnetic resonance spectra were recorded in dimethyl sulfoxide-d_6 using a JEOL Ltd. ECZ-500 spectrometer (500 MHz). Fourier transform infrared (FT-IR) spectra were recorded using a JASCO FT-IR600 spectrometer. Lap-shear tests were performed using an AGS-X universal testing machine (Shimadzu Corporation) at a crosshead speed of 1.0 mm/min. UV irradiation was performed using an LHPUV365/2501-00 (IWASAKI Electric Co., Ltd.). Ultraviolet-visible (UV-vis) absorption spectra were recorded using an Agilent Cary 8454 spectrophotometer. Differential scanning calorimetry (DSC) measurements were performed using a DSC 3500 Sirius instrument (NETZSCH). Contact angle measurements were performed using a Drop Master DMe-211 (Kyowa Interface Science Co., Ltd.).

2 FT-IR and UV-vis Measurements

2.1 Base Screening for the Redox Initiation System Using FT-IR Spectroscopy

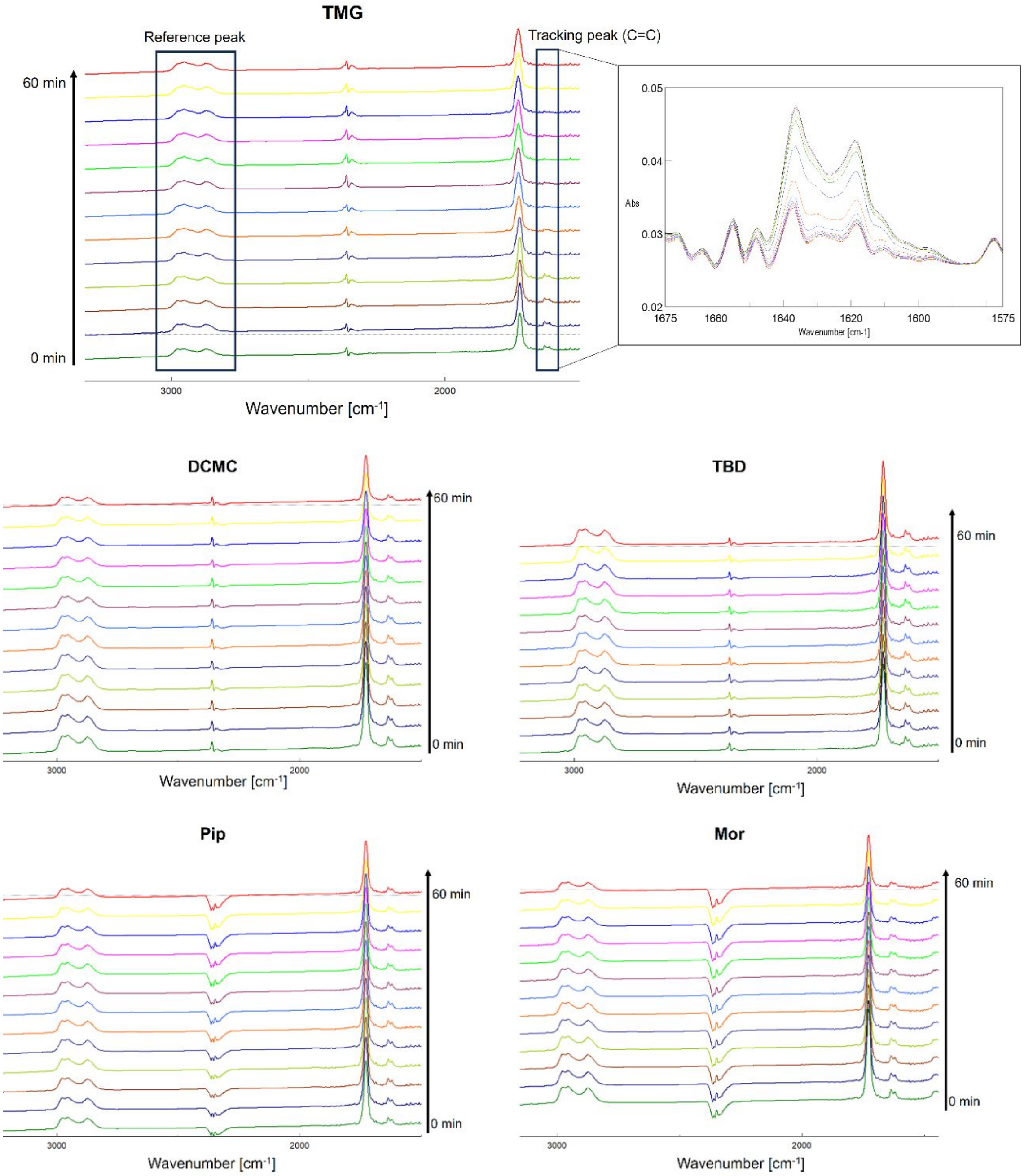

Figure S1. Time-dependent Fourier transform infrared (FT-IR) spectra of tetrahydrofurfuryl acrylate (THFA) mixtures containing organic bases (1.0 mol%) and dilauroyl peroxide (LP) (1.0 mol%) at room temperature, recorded at 5 min intervals over 60 min. The C=C stretching band at ~1635 cm^{-1} (right inset) decreases over time, indicating polymerization of the acrylate. The C–H stretching band at ~2930 cm^{-1} was used as an internal reference (left highlighted region).

2.2 Direct Evidence of Continuous Radical Generation by TEMPO Trapping

To verify continuous radical generation by the TMG/LP redox reaction, a THF solution containing TMG, LP, and TEMPO (molar ratio 40:20:1) was prepared and placed in a 10 mm quartz cuvette at room temperature without UV irradiation. UV-vis absorption spectra were recorded at 5 min intervals over 30 min. The gradual decrease in the characteristic TEMPO absorption at ~465 nm indicates continuous trapping of carbon-centered radicals generated by the TMG/LP system.

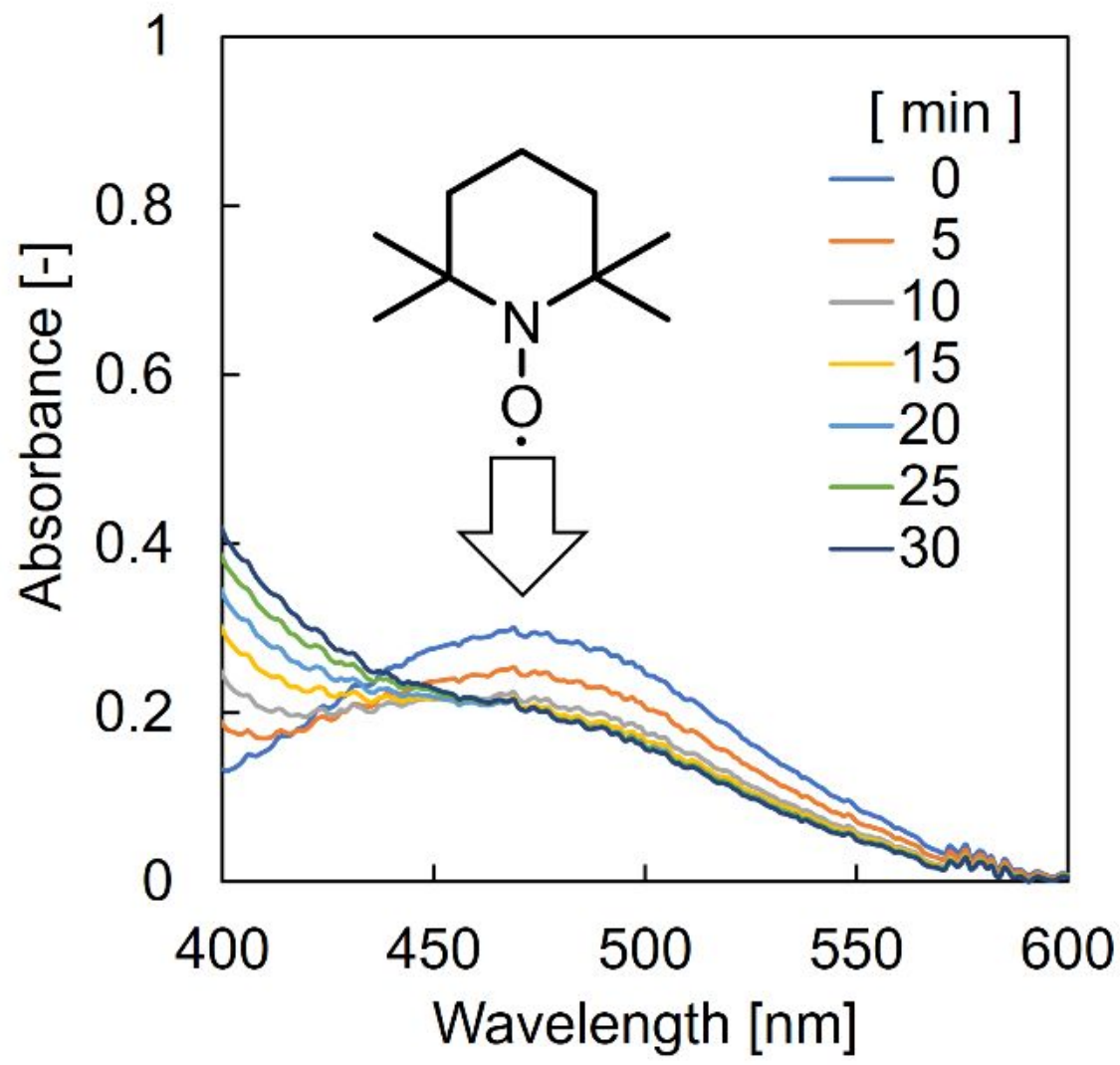

Figure S2. Time-dependent Ultraviolet-visible (UV-vis) absorption spectra of a 1,1,3,3-Tetramethylguanidine (TMG)/LP/2,2,6,6-tetramethylpiperidine 1-oxyl mixture (40:20:1 molar ratio) in THF at room temperature, recorded at 5 min intervals over 30 min.

2.3 Effects of Cou–TMG Loading on Polymerization Behavior

A solution containing the components was sandwiched between two CaF_2 substrates with a 10 μm spacer, prebaked at 60 °C for 1 min, and the FT-IR spectrum before UV exposure was recorded. The sample was then irradiated with UV light at 365 nm at 100 mW/cm^2 for 10 s (total dose of 1.0 J/cm^2) and left at room temperature, with FT-IR spectra recorded at 5 min intervals over 60 min. C=C conversion was determined by tracking the absorbance of the acrylate C=C stretching band at ~1635 cm^{-1}, normalized against the C–H stretching band at ~2930 cm^{-1}, which was used as an internal reference.

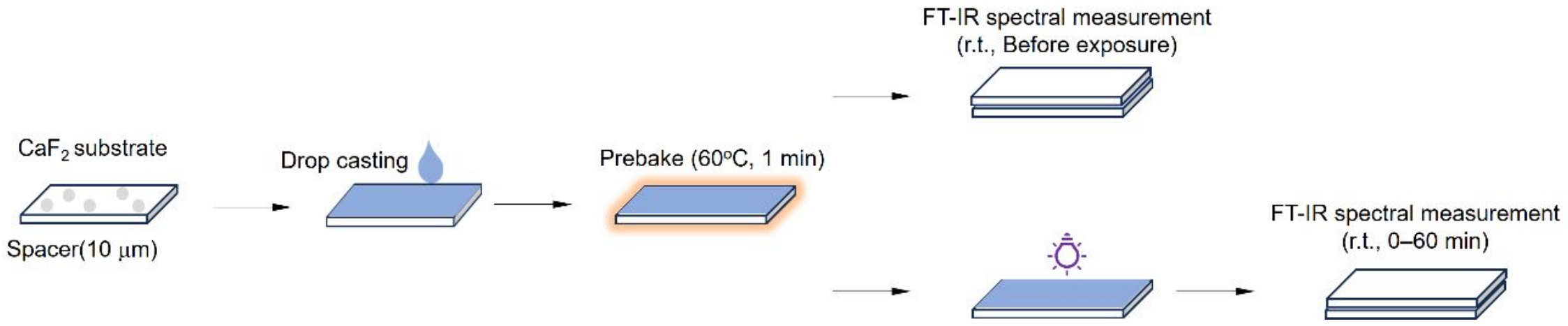

Figure S3. Schematic of the FT-IR measurement procedure.

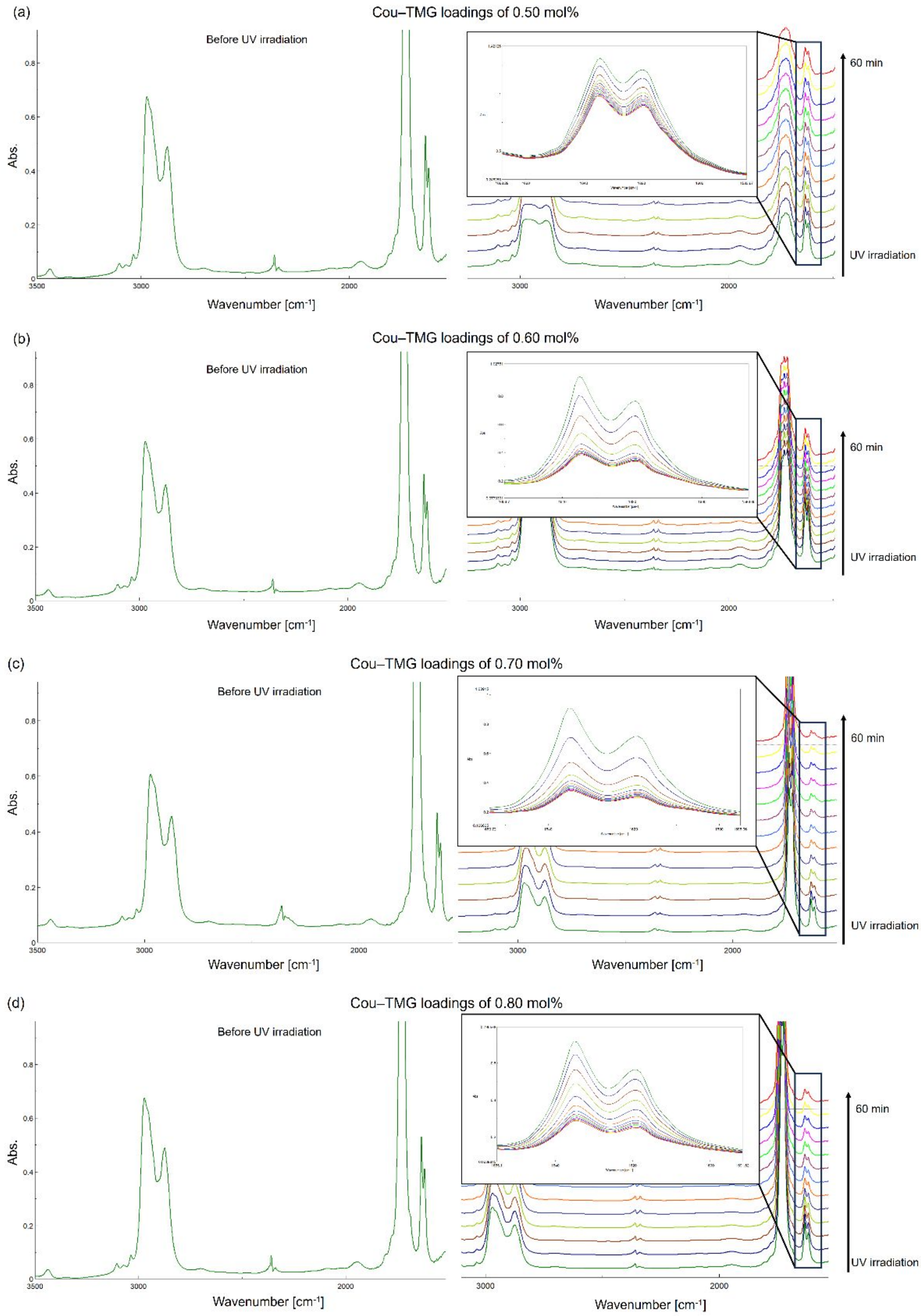

Figure S4. Time-dependent FT-IR spectra of THFA/trimethylolpropane triacrylate (TMPTA) (1:1 C=C molar ratio) mixtures containing LP (0.80 mol%) and Cou–TMG at the loadings of (a) 0.50 mol%, (b) 0.60 mol%, (c) 0.70 mol%, and (d) 0.80 mol% cast onto CaF_2 substrates.

2.4 Effect of open time on delayed-onset UV curing

To evaluate the practical workability of the resin after UV irradiation, the resin formulation was applied onto an aluminum (Al) substrate and irradiated with UV light at 365 nm (100 mW/cm², 1.0 J/cm²). After irradiation, the resin was left exposed to air at room temperature for 10 s, 30 s, 1 min, 5 min, 10 min, 20 min, 30 min, or 60 min, and its physical state, including fluidity and apparent viscosity, was visually examined (Figure S5). A second Al substrate was then laminated onto the irradiated resin and subsequently separated, and the transfer of the adhesive resin onto the opposing substrate was visually evaluated.
For FT-IR analysis, the resin formulation was irradiated with UV light at 365 nm (100 mW/cm², 1.0 J/cm²) and left exposed to air at room temperature for 10, 30, 60, or 120 s. The irradiated resin was then sandwiched between two CaF_2 substrates, and FT-IR spectra were recorded 60 min after lamination to determine the C=C conversion.

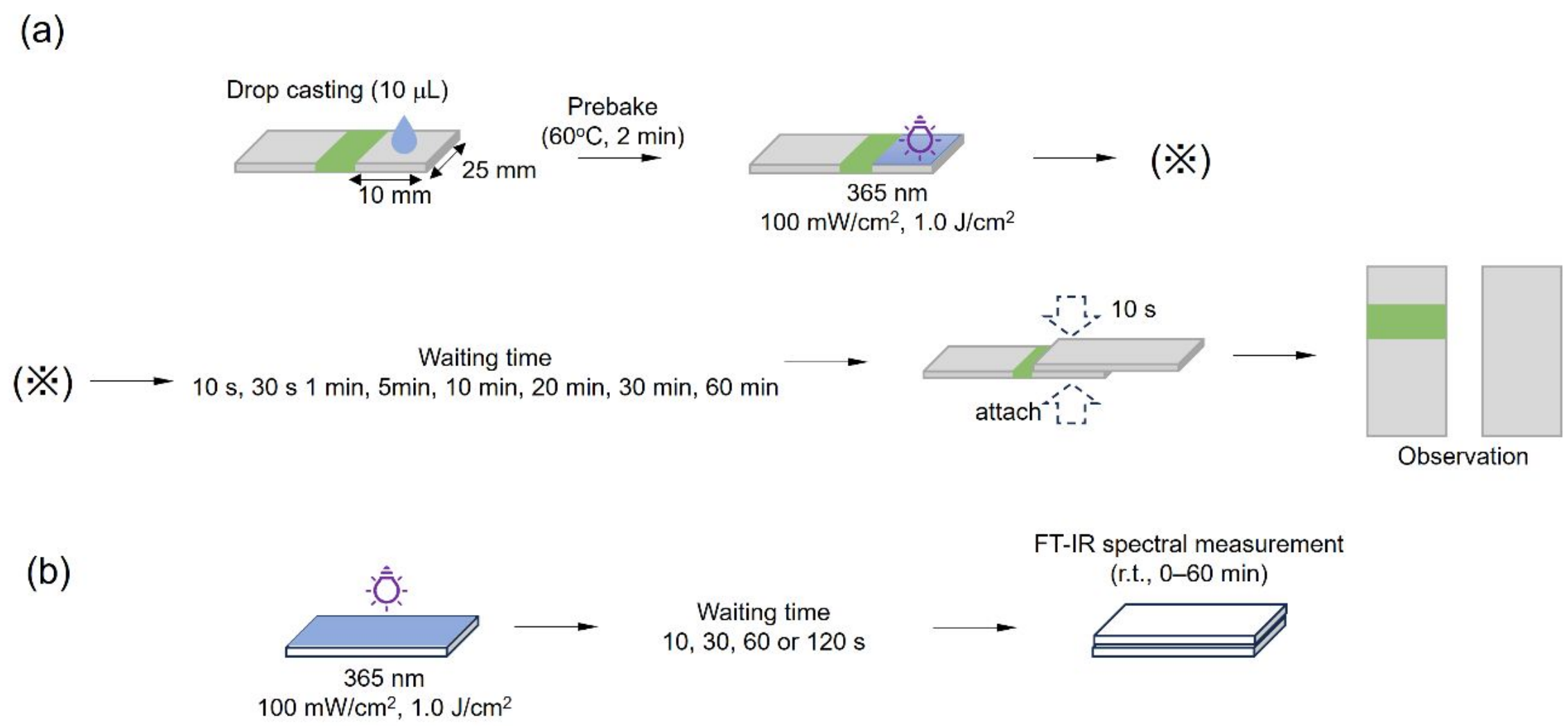

Figure S5. Evaluation of the practical open time of the adhesive resin after UV irradiation. (a) Photographs of the adhesive resin on Al substrates after standing for 10 s, 30 s, 1 min, 5 min, 10 min, 20 min, 30 min, and 60 min following UV irradiation (365 nm, 100 mW/cm², 1.0 J/cm²), and photographs of the opposing Al substrates after lamination and subsequent separation. (b) C=C conversion determined by FT-IR spectroscopy 60 min after lamination at different open times (10, 30, 60, and 120 s) following UV irradiation.

Table S1. Visual evaluation of the fluidity and transfer behavior of the adhesive resin after UV irradiation at different standing times.

Open time	10 s	30 s	1 min	5 min	10 min	20 min	30 min	60 min
Before lamination	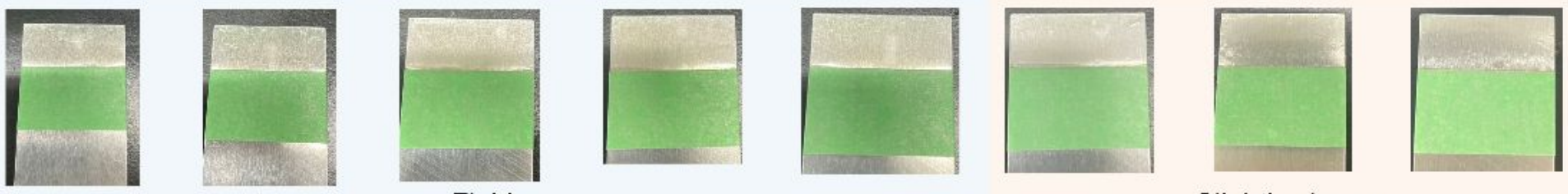							
			Fluid				Slightly viscous	
After lamination and separation	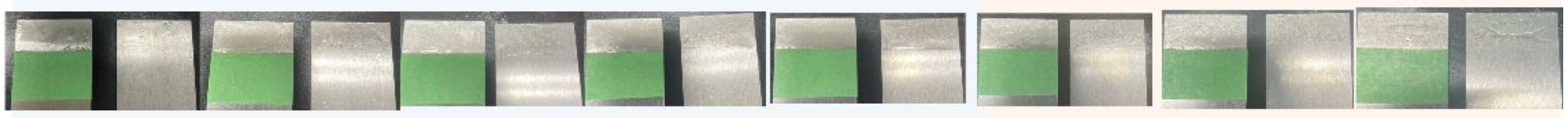							
			Resin transfer				Slight resin transfer	

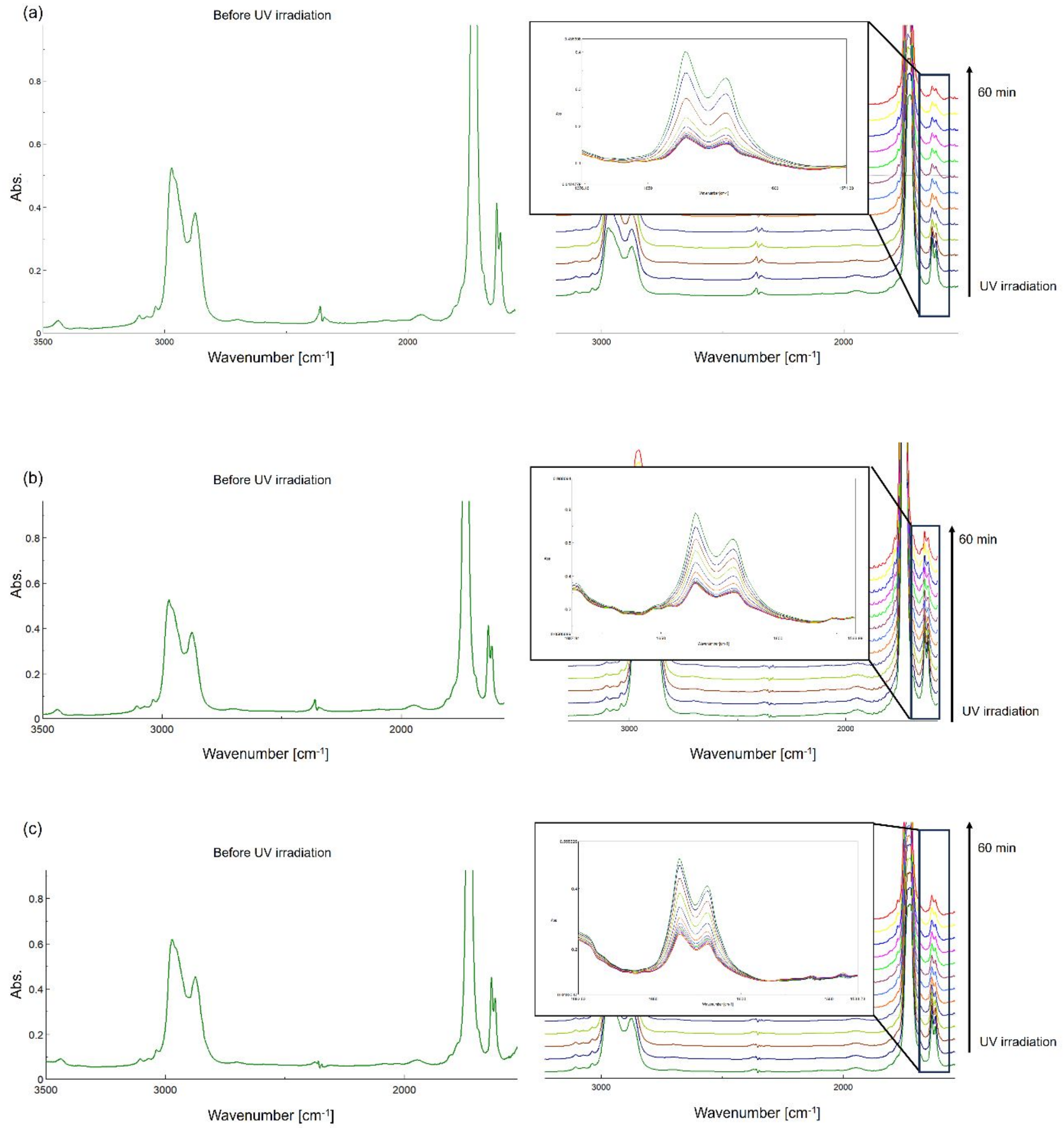

Figure S6. FT-IR spectra of THFA/TMPTA (1:1 C=C molar ratio) mixtures containing Cou–TMG (0.70 mol%) and LP (0.80 mol%) cast onto CaF_2 substrates and irradiated at 365 nm (100 mW/cm²) with a total UV dose of 1.0 J/cm². After irradiation, the samples were left exposed to air at room temperature for open times of (a) 30 s, (b) 60 s, and (c) 120 s before lamination. FT-IR spectra were recorded 60 min after lamination.

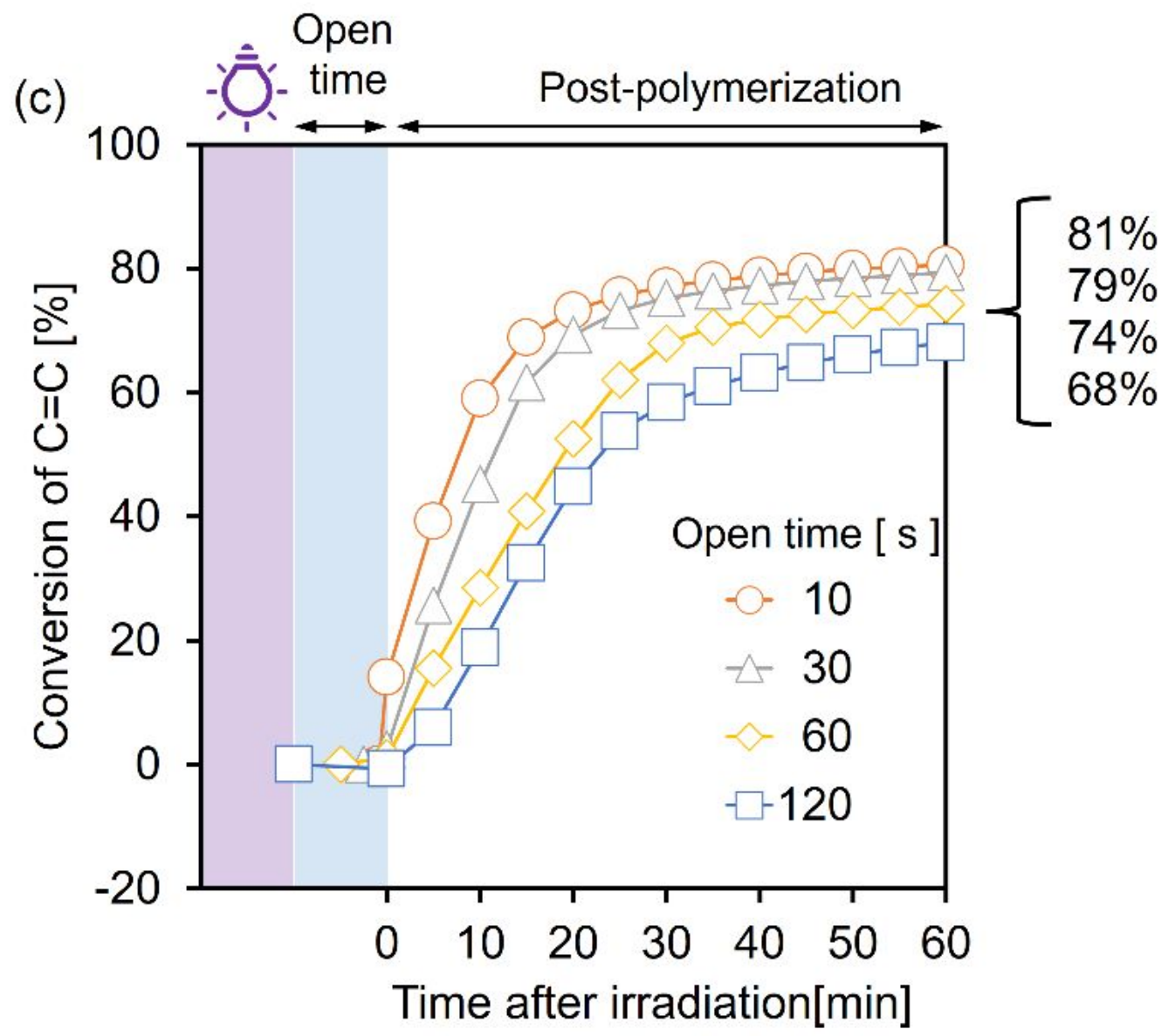

Figure S7. Final C=C conversion after 60 min for different open times (10, 30, 60, and 120 s) between UV irradiation and substrate lamination.

2.5 Control experiment in the absence of LP

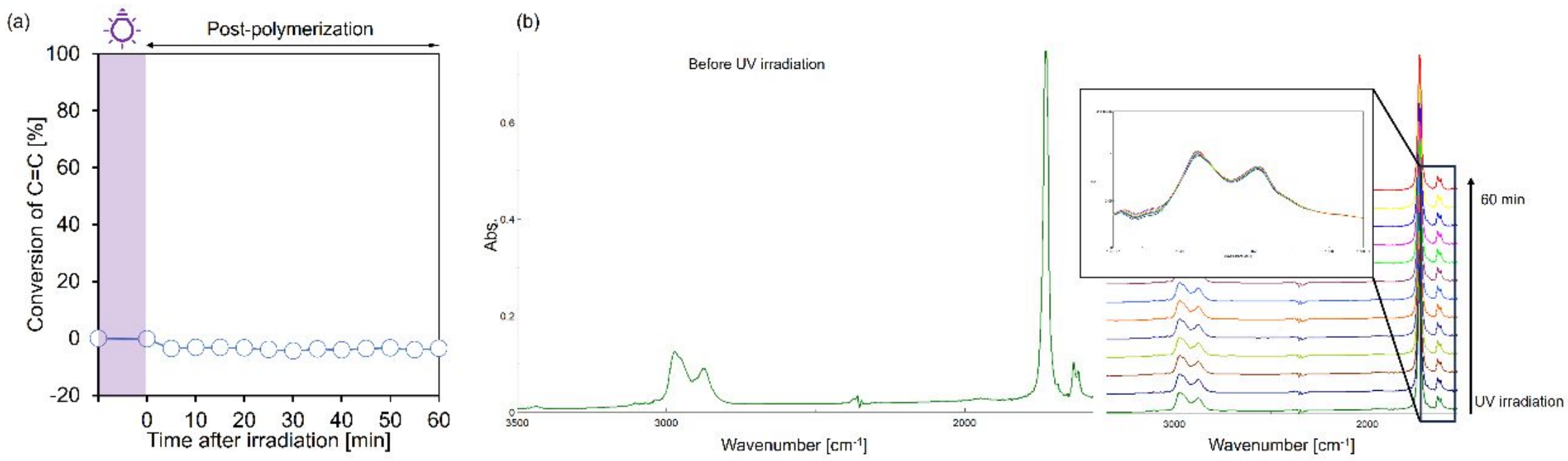

Figure S8. Control experiment in the absence of LP. (a) Time-dependent C=C conversion and (b) corresponding FT-IR spectra of the Cou–TMG/THFA/TMPTA system after UV irradiation in the absence of LP.

2.6 Comparison with a Conventional Photoradical Initiator (Omnirad 819)

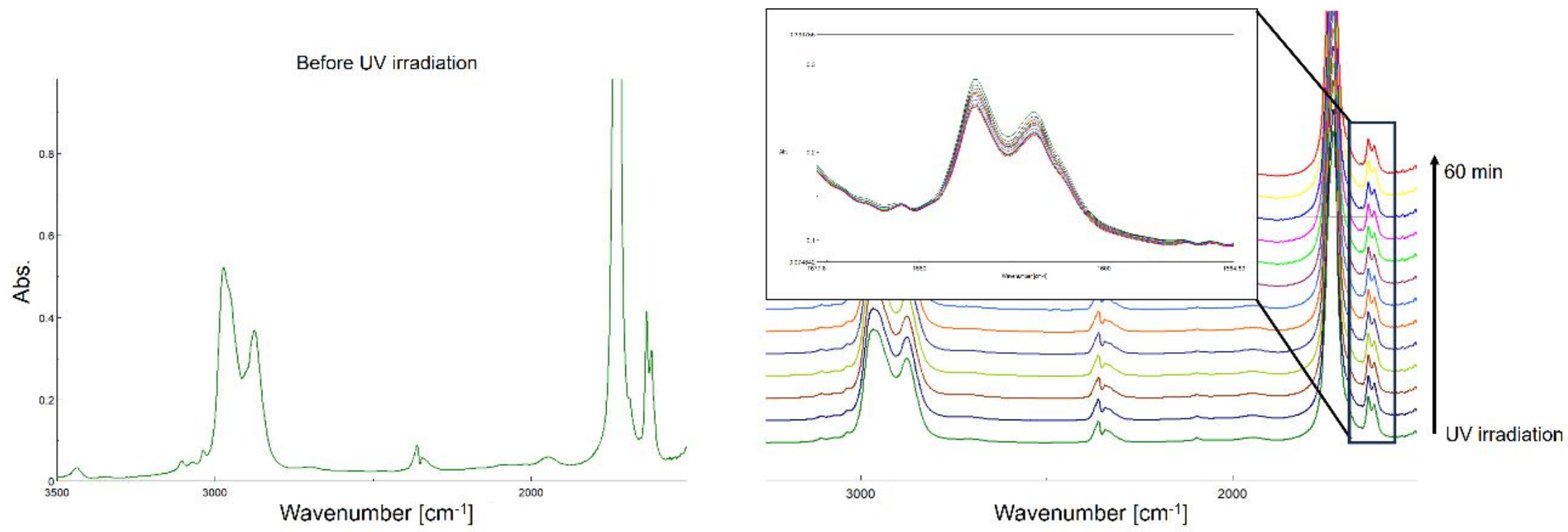

Figure S9. Time-dependent FT-IR spectra of a THFA/TMPTA (1:1 C=C molar ratio) mixture containing the conventional photoradical initiator Omnirad 819 (1.0 mol%) cast onto a CaF_2 substrate, recorded at 5 min intervals over 60 min following UV irradiation (365 nm, 100 mW/cm^2, 1.0 J/cm^2).

2.7 Effects of UV Exposure Dose on Polymerization Behavior

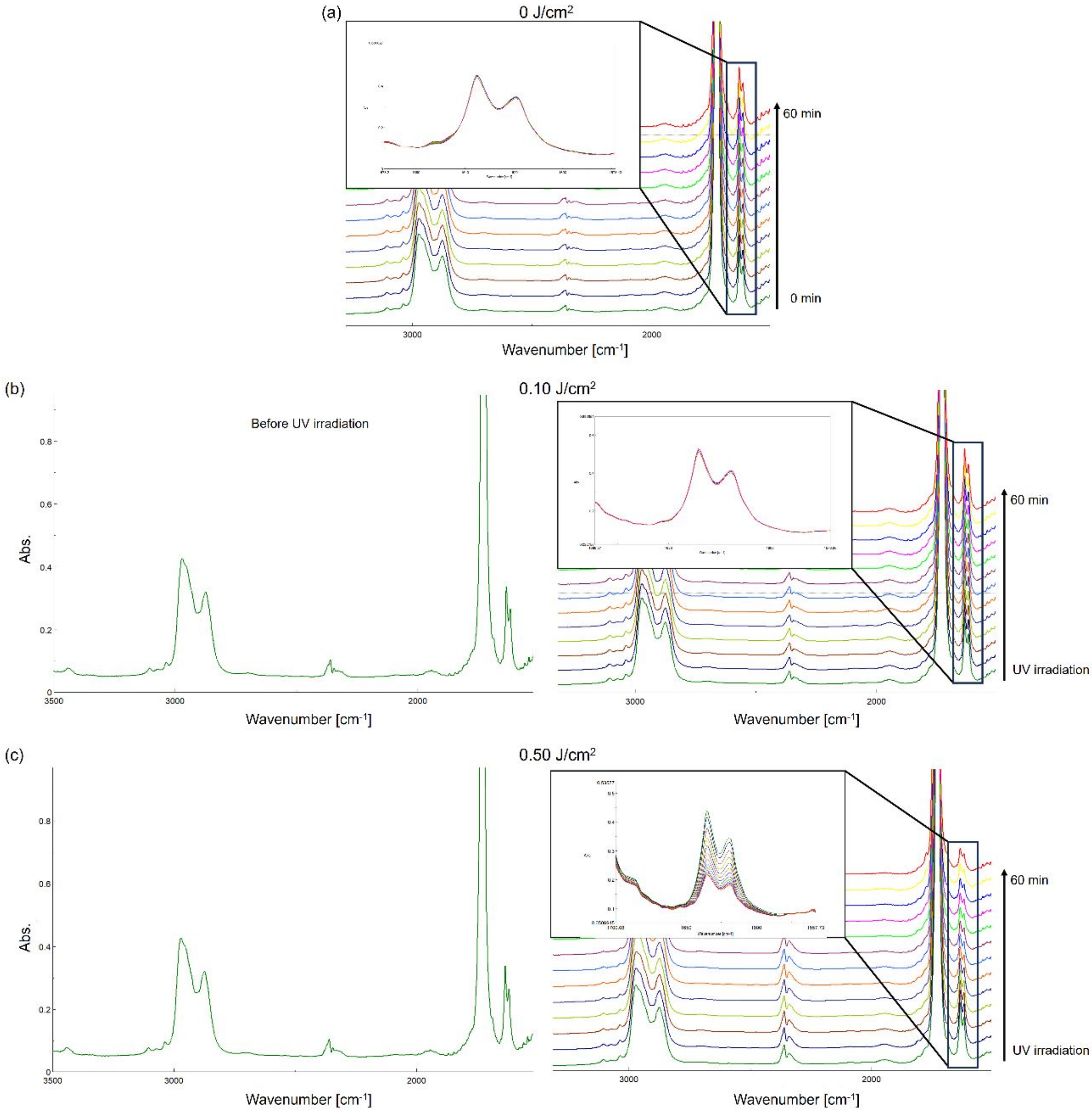

Figure S10. Time-dependent FT-IR spectra of THFA/TMPTA (1:1 C=C molar ratio) mixtures containing Cou–TMG (0.70 mol%) and LP (0.80 mol%) cast onto CaF_2 substrates and irradiated at 365 nm (100 mW/cm²) with total UV doses of (a) 0 J/cm^2 (no irradiation), (b) 0.10 J/cm^2, and (c) 0.50 J/cm^2. Spectra were recorded at 5 min intervals over 60 min following irradiation.

2.8 Polymerization Behavior of Various Multifunctional Acrylates

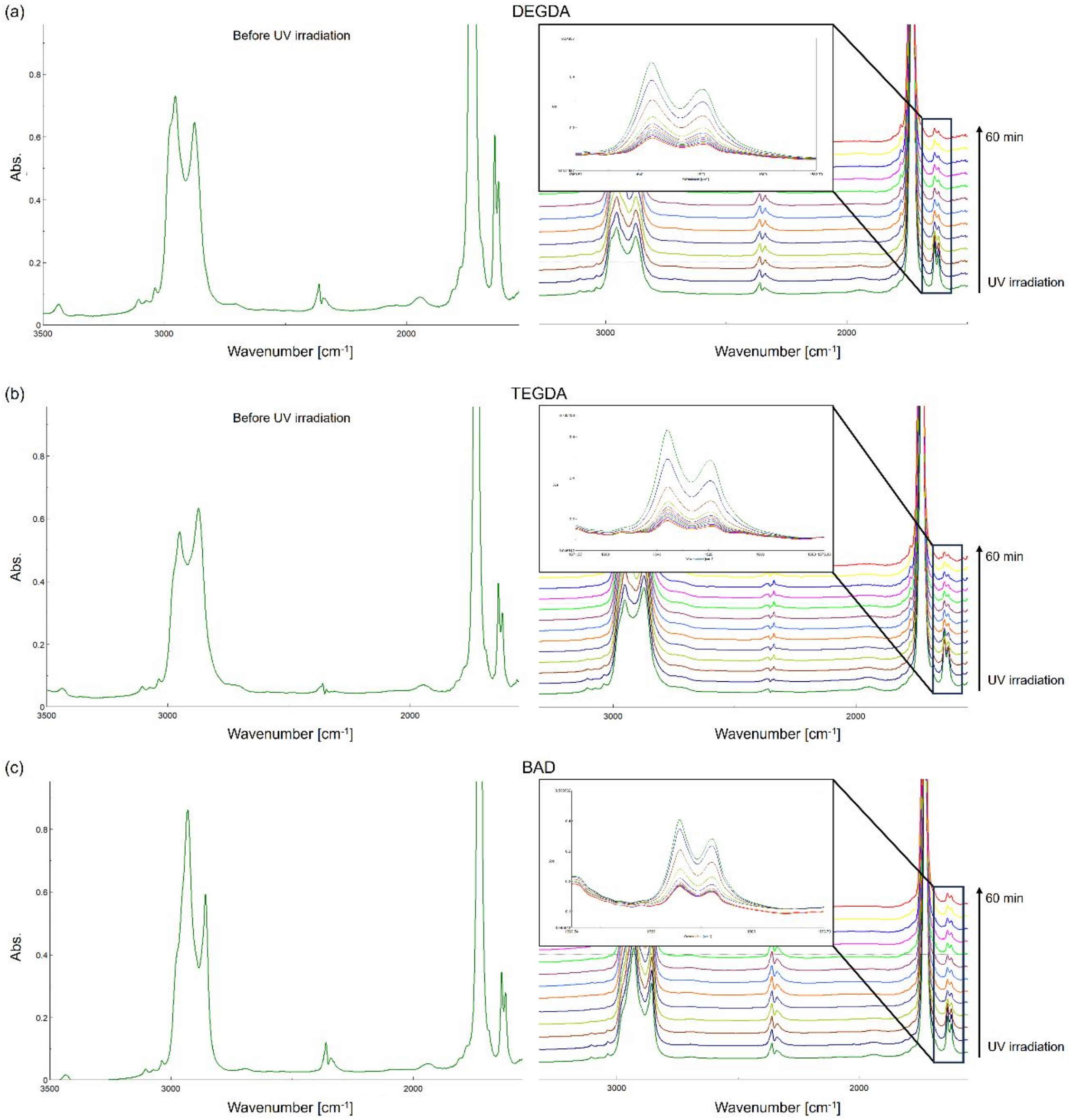

Figure S11. Time-dependent FT-IR spectra of acrylic resin mixtures cast onto CaF_2 substrates containing Cou–TMG (0.70 mol%) and LP (0.80 mol%), which consist of THFA combined with (a) diethylene glycol diacrylate (DEGDA), (b) TEGDA, or (c) 1,10-bis(acryloyloxy)decane (BAD) (1:1 C=C molar ratio). Spectra were recorded at 5 min intervals over 60 min following UV irradiation (365 nm, 100 mW/cm^2, 1.0 J/cm^2).

3 Surface Treatment of Aluminum Substrates

The substrates used for adhesion were immersed in a TSA solution in a Petri dish and sonicated at room temperature for 20 min. The substrates were then dried under reduced pressure at 100 °C for 1 h, followed by degreasing with methanol to generate surface-treated substrates. The decrease in contact angle confirms successful immobilization of the silane coupling agent on the aluminum surface.

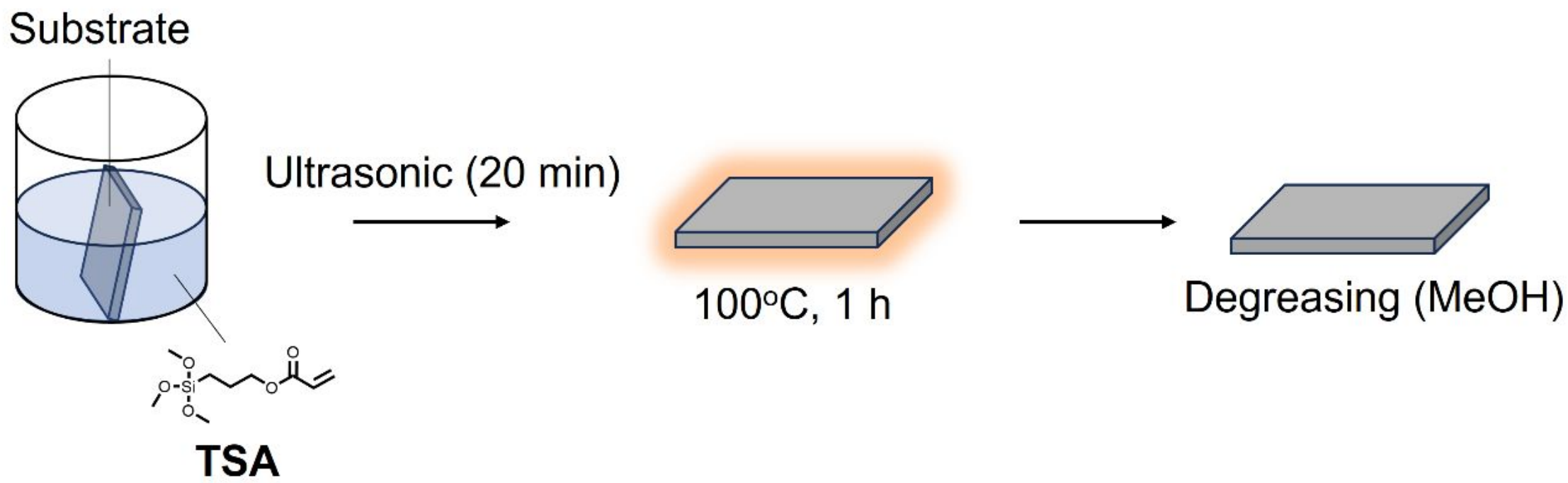

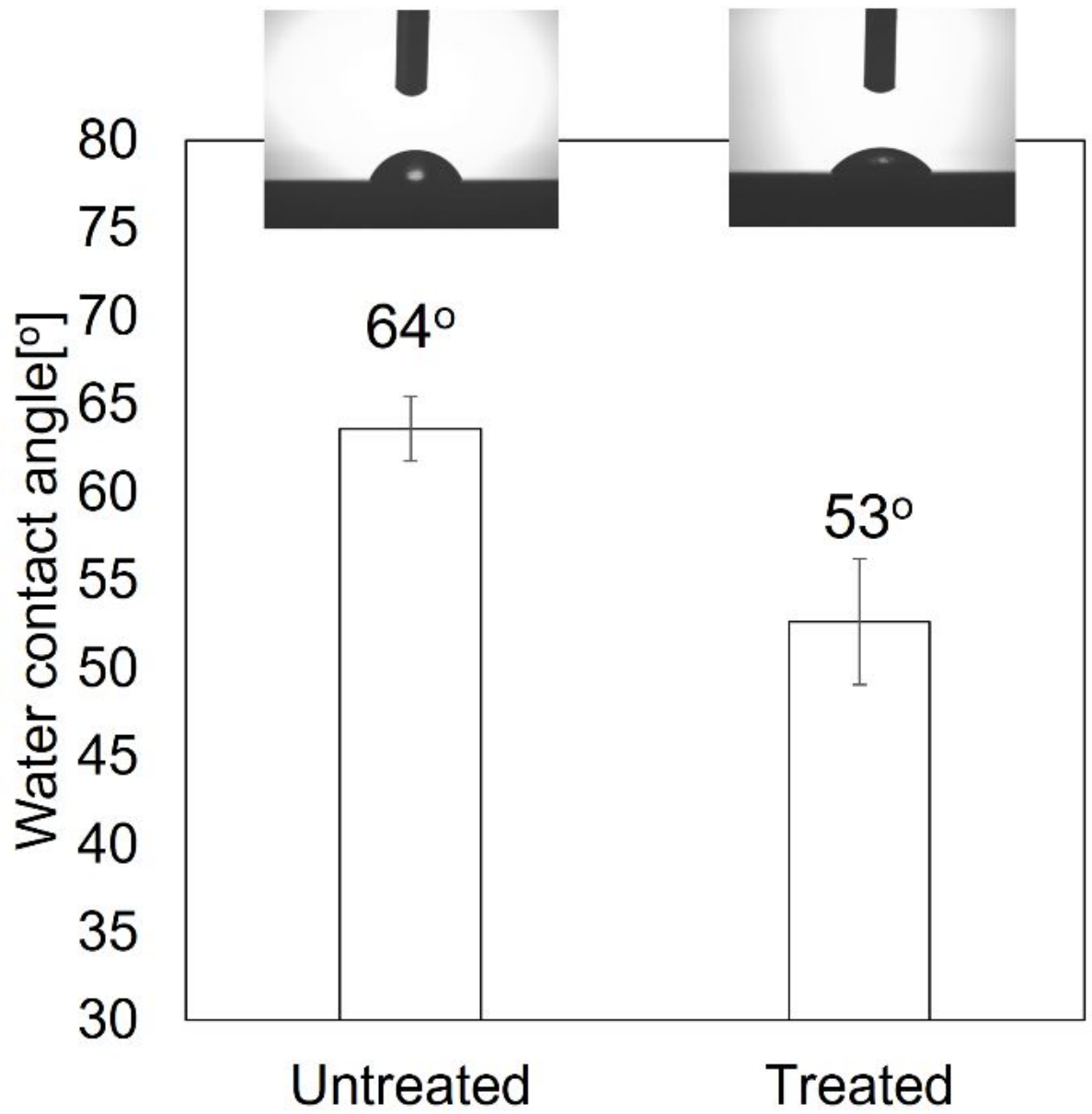

Figure S12. Water contact angles of aluminum substrates before and after silane modification (N = 5).

4 Lap-shear Performance of Various Multifunctional Acrylates

Adhesive strength was determined using an AGS-X universal testing machine (Shimadzu Corporation) at a crosshead speed of 1.0 mm/min. A 10 μL aliquot of the resin formulation was deposited on the 10 × 25 mm overlap region of a substrate (1.5 × 25 × 100 mm), prebaked at 60 °C for 2 min, and irradiated with UV light (365 nm, 100 mW/cm^2, 1.0 J/cm^2). A second substrate was laminated onto the irradiated resin within approximately 3 s, and the bonded specimens were left at room temperature for 1 h before lap-shear testing.

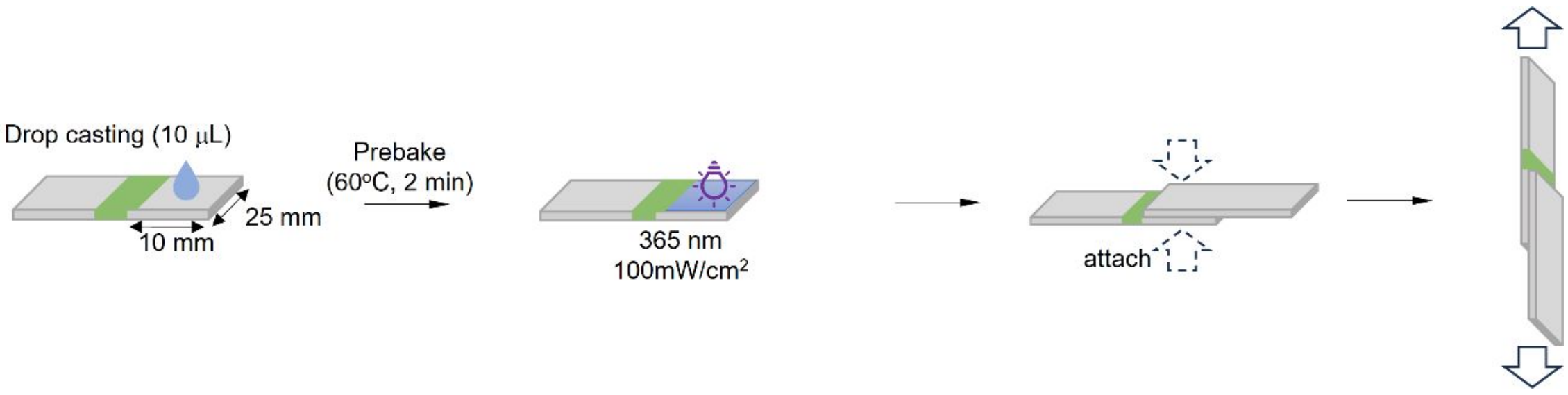

Figure S13. Schematic of the lap-shear specimen preparation and testing procedure.

Table S2. Lap-shear strength, C=C conversion, glass transition temperature (T_g), and failure mode for 3-(trimethoxysilyl)propyl acrylate (TSA)-pretreated aluminum substrates bonded with the photoinduced redox system using various multifunctional acrylates (N = 5).

Sample	Acrylate	Conversion of C=C [%]	*Tg* [ºC]	Lap shear strength (MPa)	Std. dev	Failure mode
1	**THFA TMPTA**	81	18	4.4	0.78	Interfacial failure
2	**THFA DEGDA**	83	16	3.3	0.36	Interfacial failure
3	**THFA TEGDA**	90	3.0	1.3	0.34	Interfacial failure
4	**THFA BAD**	78	1.8	1.3	0.23	Interfacial failure

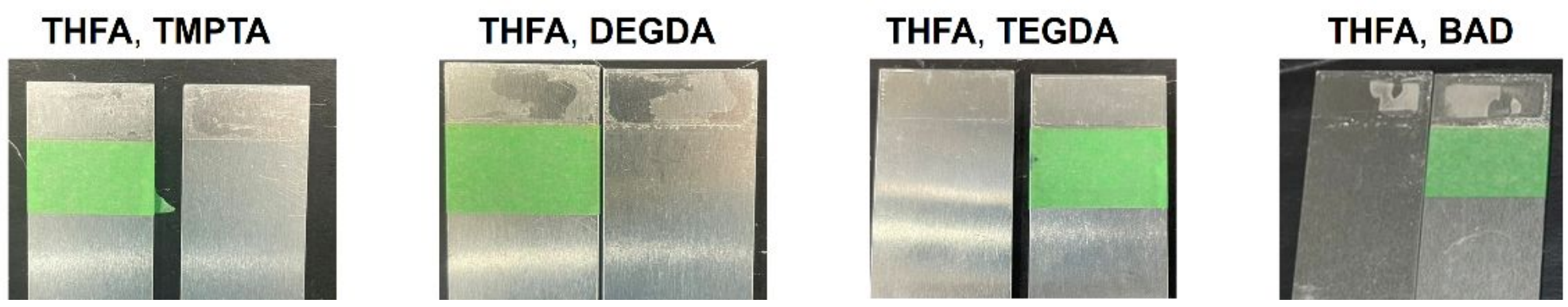

5 Surface Treatment of Other Substrates (Cu, Fe, Al, and Glass)

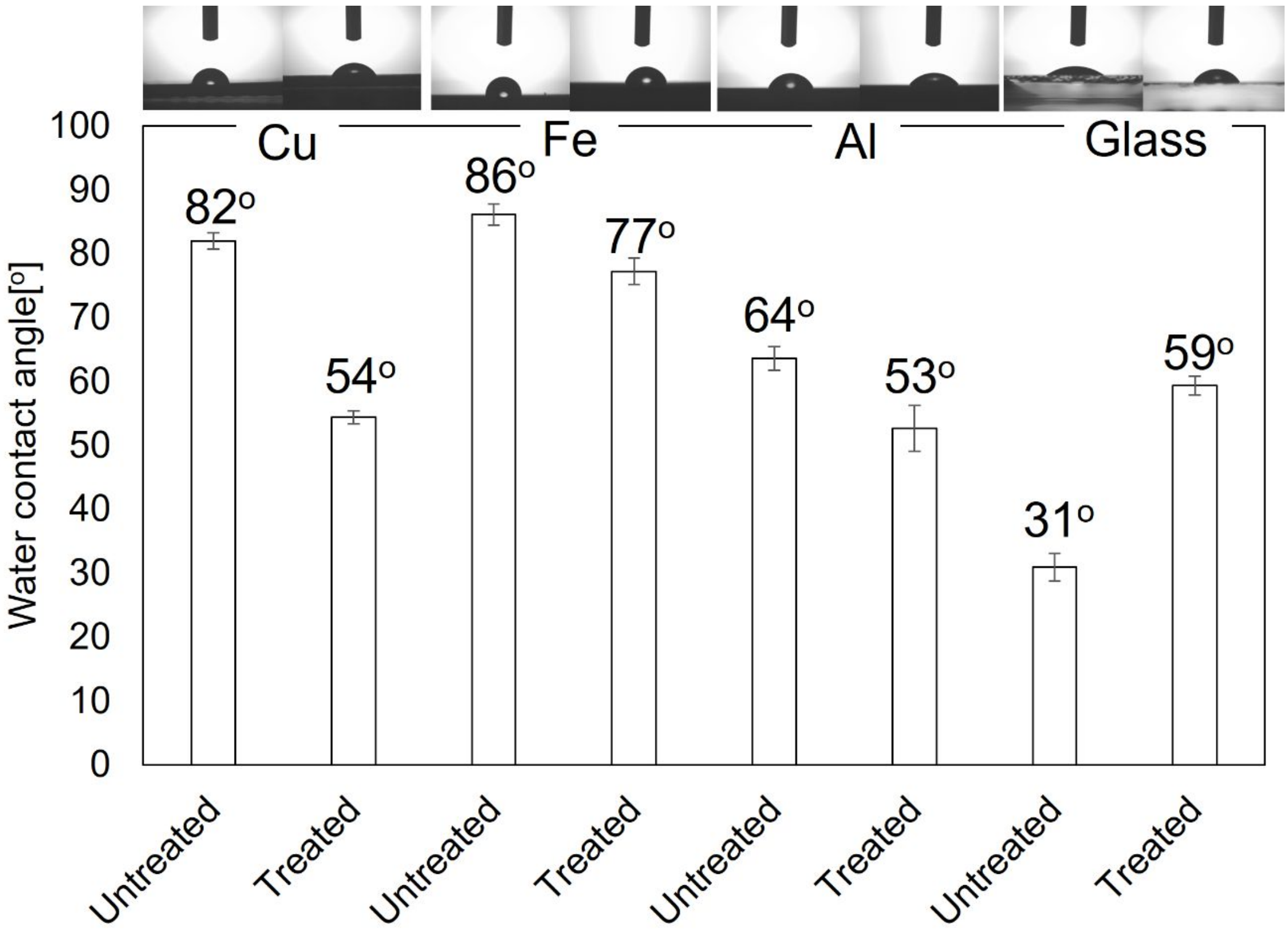

Figure S14. Water contact angles of Cu, Fe, Al, and glass substrates before (untreated) and after (treated) silane surface modification with TSA (N = 5).

6 Adhesion Performance of the THFA/TMPTA System on Various Substrates

Table S3. Lap-shear strength and failure mode of TSA-pretreated Cu, Fe, Al, polycarbonate, and glass substrates bonded with the tetrahydrofurfuryl acrylate (THFA)/trimethylolpropane triacrylate (TMPTA)-based photoinduced redox initiation system (coumarin-based 1,1,3,3-Tetramethylguanidine 0.70 mol%, dilauroyl peroxide 0.80 mol%; 1:1 THFA/TMPTA C=C molar ratio; N = 5).

Substrate	Lap shear strength (MPa)	Std. dev	Failure mode
Glass	4.8	0.62	Interfacial failure
Al	4.4	0.78	Interfacial failure
Fe	3.5	0.67	Interfacial failure
PC	3.0	0.20	Cohesive failure
Cu	1.9	0.28	Interfacial failure

7 Reference

(1) Tanaka, Y.; Aoki, D.; Arimitsu, K. Delayed-onset UV-curable Adhesives with Long-term Storage Stability by Michael Addition Reaction between Multifunctional Acrylate and Acetoacetic Acid Esters Using Photobase Generators. *Journal of Polymer Science* 2024, *62* (10), 2007–2013.
